# Unique episymbiotic relationship between *Candidatus* Patescibacteria and *Zoogloea* in activated sludge flocs at a municipal wastewater treatment plant

**DOI:** 10.1111/1758-2229.70007

**Published:** 2024-09-12

**Authors:** Naoki Fujii, Kyohei Kuroda, Takashi Narihiro, Yoshiteru Aoi, Noriatsu Ozaki, Akiyoshi Ohashi, Tomonori Kindaichi

**Affiliations:** ^1^ Department of Civil and Environmental Engineering, Graduate School of Advanced Science and Engineering Hiroshima University Hiroshima Japan; ^2^ Bioproduction Research Institute National Institute of Advanced Industrial Science and Technology (AIST) Sapporo Japan; ^3^ Program of Biotechnology, Graduate School of Integrated Sciences for Life Hiroshima University Hiroshima Japan

## Abstract

*Candidatus* Patescibacteria, also known as candidate phyla radiation (CPR), including the class‐level uncultured clade JAEDAM01 (formerly a subclass of Gracilibacteria/GN02/BD1‐5), are ubiquitous in activated sludge. However, their characteristics and relationships with other organisms are largely unknown. They are believed to be episymbiotic, endosymbiotic or predatory. Despite our understanding of their limited metabolic capacity, their precise roles remain elusive due to the difficulty in cultivating and identifying them. In previous research, we successfully recovered high‐quality metagenome‐assembled genomes (MAGs), including a member of JAEDAM01 from activated sludge flocs. In this study, we designed new probes to visualize the targeted JAEDAM01‐associated MAG HHAS10 and identified its host using fluorescence in situ hybridization (FISH). The FISH observations revealed that JAEDAM01 HHAS10‐like cells were located within dense clusters of *Zoogloea*, and the fluorescence brightness of zoogloeal cells decreased in the vicinity of the CPR cells. The *Zoogloea* MAGs possessed genes related to extracellular polymeric substance biosynthesis, floc formation and nutrient removal, including a polyhydroxyalkanoate (PHA) accumulation pathway. The JAEDAM01 MAG HHAS10 possessed genes associated with type IV pili, competence protein EC and PHA degradation, suggesting a *Zoogloea*‐dependent lifestyle in activated sludge flocs. These findings indicate a new symbiotic relationship between JAEDAM01 and *Zoogloea*.

## Graphical abstract

In this study, we reconstructed the high‐quality metagenome‐assembled genome of JAEDAM01, designed new probes to visualize members of JAEDAM01 and identified its host, *Zoogloea*, using fluorescence in situ hybridization (FISH). Our findings indicate a novel symbiotic relationship between Ca. Patescibacteria and Procaryote.

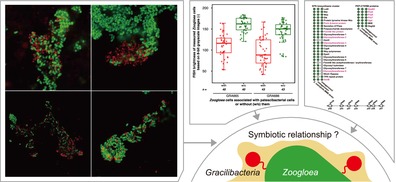

## INTRODUCTION

The activated sludge method is a biological treatment that has been widely used in wastewater treatment plants for over 100 years. This method removes organic matter and nutrients from influent wastewater through metabolic reactions of various microorganisms in activated sludge. However, the detailed taxonomic and physiological characteristics of the microbial communities within activated sludge, which are known for their complexity, are not well understood (Kindaichi et al., 2016). Among these diverse bacteria, several members of the bacterial phylum *Candidatus* Patescibacteria (also known as the candidate phyla radiation [CPR] superphylum), a large and diverse bacterial group consisting of uncultivated bacteria, are universally present (Fujii et al., 2022; Kagemasa et al., 2022; Kindaichi et al., 2016; Zhang et al., 2012). Ca. Patescibacteria are characterized by their limited metabolic potential, as inferred from metagenomic analyses (Albertsen et al., 2013; Fujii et al., 2022; Wrighton et al., 2012). Furthermore, microscopic observation using techniques such as fluorescence in situ hybridization (FISH) and transmission electron microscopy (TEM) have revealed that most Ca. Patescibacteria have small cell sizes. Recent studies have highlighted that Ca. Patescibacteria parasitize other bacterial or archaeal microorganisms, suggesting a lifestyle that may compensate for their metabolic deficiencies (He et al., 2015; Kuroda, Yamamoto, et al., 2022; Kuroda, Kubota, et al., 2022; Moreira et al., 2021; Xie et al., 2022; Yakimov et al., 2022). Within the Ca. Patescibacteria phylum, Ca. Saccharimonadia (formerly TM7), Ca. Paceibacteria (formerly Parcubacteria/OD1) and JAEDAM01 (formerly subclass of Gracilibacteria/GN02/BD1‐5) are commonly detected in the activated sludge flocs (Albertsen et al., 2013; Fujii et al., 2022; Kindaichi et al., 2016; Singleton et al., 2021). Recently, JAEDAM01 has also been detected in lake water and information has been obtained by FISH, SEM and TEM (Moreira et al., 2021; Yakimov et al., 2022); however, the information is limited when compared to the other members of *Patescibacteria*, such as *Saccharimonadia*. To better understand microbial interactions in activated sludge flocs, in situ, visualization using FISH or TEM within the flocs is very important because FISH probes specific for JAEDAM01 did not exist until recently. In this study, we designed new probes based on full‐length 16S rRNA genes from previously obtained metagenome‐assembled genomes (MAGs) related to JAEDAM01 from activated sludge flocs and visualized them using FISH. The FISH observations showed that JAEDAM01 surrounds *Zoogloea* cells in activated sludge flocs, indicating a symbiotic metabolic interaction. Therefore, the metabolic potentials and global distributions of JAEDAM01 and *Zoogloea* in activated sludge flocs were predicted using the MAGs recovered in a previous study (Fujii et al., 2022) and a previously reported global survey of 16S rRNA gene amplicon sequencing in wastewater treatment plants (Hu et al., 2024).

## EXPERIMENTAL PROCEDURES

### Sample collection

As previously described (Fujii et al., 2022), activated sludge samples were collected from aeration tanks in a wastewater treatment plant in Higashihiroshima City in February 2019 (designated as AS201902) and April 2020 (designated as AS202004). The AS202004 sample was anaerobically incubated for 3 days to change the relative abundance of Ca. Patescibacteria and designated as AA202004. Fresh and incubated sludge samples were stored at −18°C for further analyses.

### 16S rRNA gene amplicon sequences and metagenomic analysis

The 16S rRNA gene amplicon sequencing results were obtained from a previous study (Fujii et al., 2022) using QIIME2 v2021.11 (Bolyen et al., 2019) and the SILVA v138.1 database (Quast et al., 2013). Full‐length 16S rRNA gene sequences of *Zoogloea* were reconstructed using EMIRGE software (–151‐i 350‐s 50‐phred33) (Miller et al., 2011). The 16S rRNA gene sequence was compared with the partial length of amplicon sequence variants (ASVs) of *Zoogloea* (Table S1). The partial sequence of the most abundant zoogloeal ASV corresponded to the reconstructed 16S rRNA gene with 100% identity (Figure S1A,B). The phylogenetic tree of 16S rRNA gene sequences of JAEDAM01 and *Zoogloea* were constructed based on neighbour‐joining methods in ARB software v7.0 (Ludwig et al., 2004) using the SILVA138.1 database for small subunit rRNA gene sequences from 1000 resamplings. Thermotoga sequences were used as an outgroup. MAGs of JAEDAM01 and *Zoogloea* (DRA013531) were obtained from a previous study (Fujii et al., 2022). The phylogenetic classification of *Zoogloea* was evaluated using GTDB‐Tk v2.2.6 (Chaumeil et al., 2019). The completeness and contamination of the bins were assessed using CheckM v1.1.2 (Parks et al., 2015). Genomes were annotated using a combination of Prokka v1.130 (Seemann, 2014), GhostKOALA (Kanehisa et al., 2016), DRAM v1.2.3 (Shaffer et al., 2020) and manual annotation. Genetic codes were examined with Codetta v2.0 (Shulgina & Eddy, 2023). The genome tree was constructed using a combination of GTDB‐Tk and IQ‐TREE v2.2.2.3. Conserved marker genes were identified using ‘gtdbtk identify’ as the default parameter, and a phylogenetic filter (–taxa_filter f__Rhodocyclaceae or c__Gracilibacteria, c__JAEDAM01) was applied with ‘gtdbtk align’ and aligned to the reference genome using ‘gtdbtk align’. Phylogenetic trees were constructed using an auto‐optimized surrogate model (Q.insect + F + R10 or LG + F + I + R10) in IQ‐TREE (B 1000) (Minh et al., 2020). The two reconstructed *Zoogloea* MAGs did not contain the 16S rRNA gene; thus, they were classified into the genus *Zoogloea* in a phylogenetic tree based on concatenated phylogenetic marker genes (Figure S4A). Therefore, we speculated that both the reconstructed full‐length 16S rRNA gene and genomes were derived from dominant *Zoogloea* in activated sludge flocs. Zoogloeal MAGs were assigned to the reference genome of *Zoogloea resiniphila* MMB strain (KX259245) (An et al., 2016) using BLASTp v2.6.0 (Altschul et al., 1990) for manual annotation. To investigate the diversity and global abundance of JAEDAM01‐Zoogloea association in wastewater treatment plants, raw 16S rRNA gene amplicon sequencing datasets (V4 region) were obtained from the NCBI repository under BioProject accession number PRJNA1013122 (Hu et al., 2024). The datasets include 1083 Sequence Read Archive (SRA) sequence data under 542 BioSample datasets. The 16S rRNA gene sequences were trimmed, screened and assembled using DADA2 (Callahan et al., 2016) under QIIME2 (Bolyen et al., 2019). After processing with DADA2, 14 samples that had less than 1000 reads were excluded from the microbiome analysis of this study (datasheet 1 in Data S2). Operational taxonomic units (OTUs) were clustered with a sequence identity cut‐off of ≥97% using VSEARCH (Rognes et al., 2016). Taxonomic classification was performed using classify‐sklearn with the SILVA database version 138 (Quast et al., 2013) and Greengenes2 (McDonald et al., 2023). Representative OTUs of JAEDAM01 and Zoogloea were selected with a sequence identity cut‐off of ≥97% with the 16S rRNA gene sequences of JAEDAM01 HHAS10 and Zoogloea MAGs. A total of 51 OTUs that perfectly matched the newly designed GRA686 probe (as described in the Probe design and FISH subsection) were selected to calculate the sum of relative abundances of the targeted JAEDAM01 in the activated sludge samples. Spearman's rank correlations between representative Ca. Patescibacteria‐Zoogloea associations were calculated using the Hmisc package with the rcorr function in R ver. 4.3.1 (R_Core_Team, 2018).

### Probe design and FISH

Probes specific to JAEDAM01 were designed using ARB software v7.0 (Ludwig et al., 2004). The specificity and coverage of the newly designed GRA665 and GRA686 are shown in Figure S4. The optimal formamide concentrations of GRA665 and GRA686 were 30% each, which was predicted using mathFISH (Yilmaz et al., 2011) (Figures S5 and S6, Table 1). GRA665 is a highly specific probe and can detect only one sequence with the targeted sequence in the SILVA v138.1 database. In contrast, GRA686 can detect clusters in the SILVA v138.1 database, including closely related sequences. GRA686 was predicted to match out‐of‐class sequences with a single nucleotide mismatch; however, mathFISH (Yilmaz et al., 2011) predicted that GRA686 did not hybridize to off‐target bacteria under optimal formamide concentrations (Chiriac et al., 2022) (Figure S7). Furthermore, adding a competitor probe almost completely prevents false positive hybridization; therefore, using a competitor probe is recommended (Figure S7). NON‐GRA665 and NON‐GRA686 were designed as nonsense probes for negative control (Figure S8). Sample fixation and FISH were performed as previously described (Nielsen et al., 2009); the AS202004 sample was used for FISH. The probes used in this study are listed in Table 1. Probes were labelled at the 5′ end with either Alexa488, Alexa555 or Alexa647. Simultaneous hybridization was performed using the procedure described below. Hybridization with the probe requiring higher stringency was performed first, followed by hybridization with the probe requiring lower stringency (Wagner et al., 1994). The FISH observation was performed with an LSM700 confocal laser‐scanning microscope equipped with lasers with excitation wavelengths of 488, 555 and 639 nm. For this experiment, Alexa488‐labelled GRA665 and GRA686, and Alexa647‐labelled ZOO834 were used to avoid the possibility of fluorescence overwrapping. The FISH images were converted to 8‐bit grayscale images, and the brightness of the Zoogloeal cells was measured with ImageJ v2.14.0 (Schneider et al., 2012). The brightness of the areas where *Zoogloea* forms dense clusters with JAEDAM01 was measured within the interior cells, delineated by the boundary of cells adjacent to JAEDAM01. The brightness of the areas where *Zoogloea* without JAEDAM01 was measured within the same field of view as the former. As it was confirmed that the variance between the two target groups was unequal, Welch's t‐test calculation was performed with the R version 4.3.1 (R_Core_Team, 2018).

**TABLE 1:** The List of the oligonucleotide probes used in this study. Names, targets, sequences, formamide concentrations and references of oligonucleotide probes used in this study.

Probe name	Target	Sequence (5′ → 3′)	FA (%)	Reference
GRA665	Some members of JAEDAM01	TTA CCG TTC TGC TAG CCC	30	This study
GRA686	Some members of JAEDAM01	CAA CGG ATT GCA CCC CTA	30	This study
CompGRA686	Competitor for GRA686	CAA CGG ATT TCA CCC CTA	—	This study
NON‐GRA6665	Nonsense probe for GRA665	GGG CTA GCA GAA CGG TAA	30	This study
NON‐GRA686	Nonsense probe for GRA686	TAG GGG TGC AAT CCG TTG	30	This study
ZOO834	Most members of *Zoogloea*	CTC AAT GAG TCT CCT CAC CG	50	Oshiki et al., 2008
EUB338	Most Bacteria	GCT GCC TCC CGT AGG AGT	0–50	Amann et al., 1990
EUB338II	*Planctomycetales*	GCA GCC ACC CGT AGG TGT	0–50	Daims et al., 1999
EUB338III	*Verrucomicrobiales*	GCT GCC ACC CGT AGG TGT	0–50	Daims et al., 1999
EUB338IV	Bacteria lineages not covered by probes EUB338, EUB338II and EUB338III	GCA GCC TCC CGT AGG AGT	0–50	Schmid et al., 2005

## RESULTS AND DISCUSSION

### *FISH probes for JAEDAM01 and* Zoogloea

We reconstructed one JAEDAM01 MAG HHAS10 and two *Zoogloea* MAGs from activated sludge flocs using a previous dataset (DRA013531) (Fujii et al., 2022) (Table S2). For the selection of the specific probes, the 16S rRNA gene sequence was present in the MAG of JAEDAM01 but not in those of *Zoogloea*. Therefore, it was reconstructed using EMIRGE (Miller et al., 2011). To detect JAEDAM01 in activated sludge flocs, we designed two new oligonucleotide probes (GRA665 and GRA686) (Table 1, Figure S3). To detect *Zoogloea*, a previously reported probe, ZOO834 (Oshiki et al., 2008), was used because the probe sequence perfectly matched the reconstructed 16S rRNA gene of *Zoogloea*. The probe ZOO834 covered most *Zoogloea* species (89.7% of *Zoogloea* spp. based on SILVA v138.1 taxonomy), including the reconstructed full‐length of 16S rRNA gene sequence of *Zoogloea* in this study (Figure S1A,B).

### *In situ detection and morphology of JAEDAM01 and* Zoogloea

**FIGURE 1:**
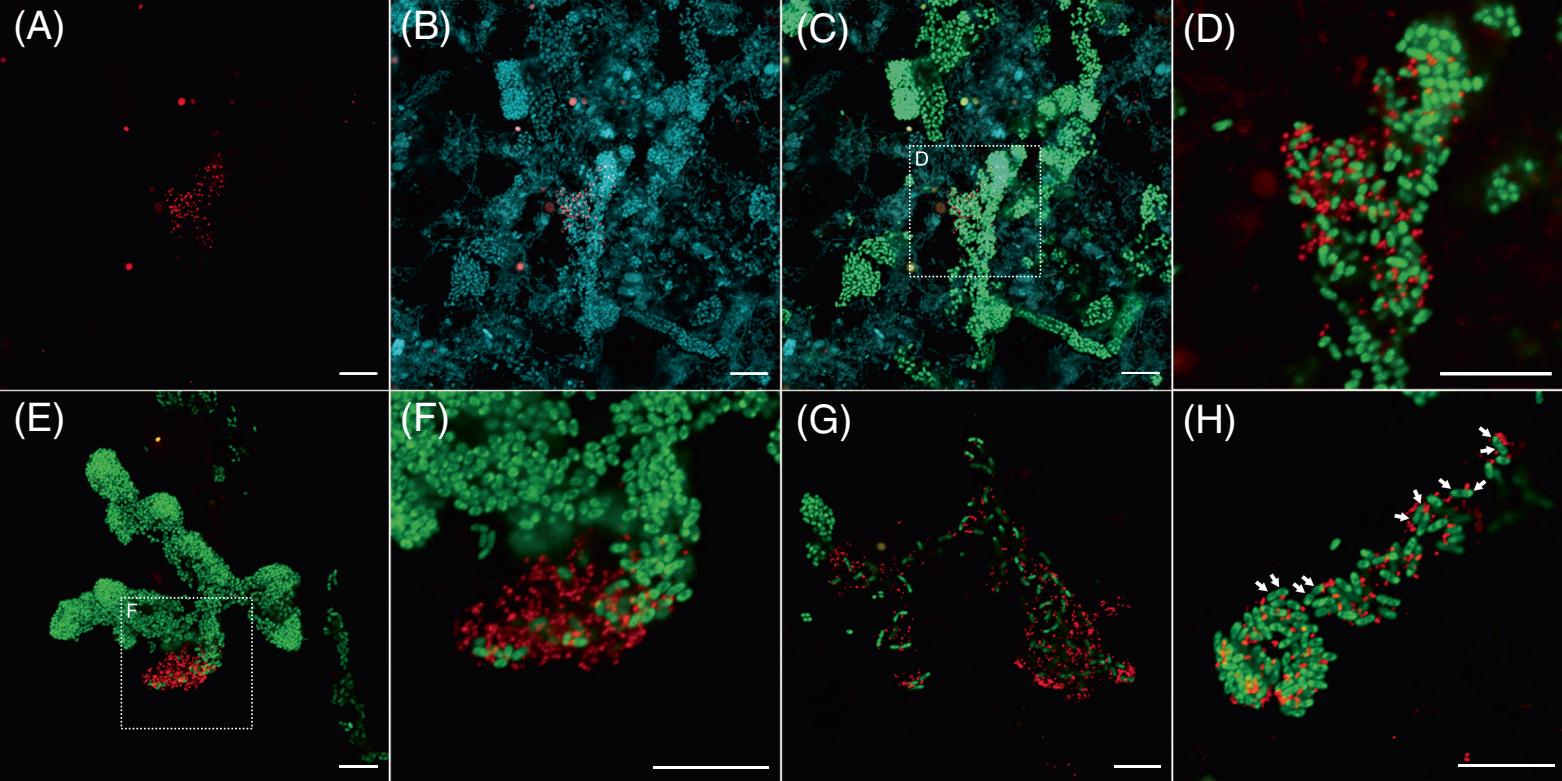
Fluorescence in situ hybridization (FISH) micrographs of JAEDAM01 and *Zoogloea* in activated sludge flocs. FISH was performed with three different fluorophores, the Alexa 488‐labelled ZOO834 probe (green), the Alexa 555‐labelled GRA655 or GRA686 probe (red) and the Alexa 647‐labelled EUBmix probes (blue). (A–C) FISH micrographs of the same field of view; (A) GRA655 (red); (B) GRA655 (red) and EUBmix (blue); (C) ZOO834 (green), GRA655 (red) and EUBmix (blue); (D) ZOO834 (green) and GRA655 (red). (E and F) ZOO834 (green) and GRA686 (red). (G and H) ZOO834 (green) and GRA655 (red). White arrows in (H) indicate zoogloeal cells undergoing cell division. Scale bars = 10 μm.

The cells hybridized with probes GRA665 and GRA686 showed loosely associated clusters of tiny cocci (<0.5 μm in diameter) (Figure 1A). These cells were also hybridized with bacteria‐specific probes, EUBmix (Figure 1B); therefore, the cells positive for probes GRA665 and GRA686 are likely to be HHAS10. The fluorescence of GRA665 and GRA686 always overlaps, confirming that each probe can detect the same target (Figure S9). HHAS10 formed loosely associated clusters within aggregates of rod‐shaped cells with dense clusters that hybridized with the EUBmix probe (Figure 1B). The rod‐shaped cells were likely *Zoogloea* spp. based on their unique finger‐like‐shaped clusters. The rod‐shaped cells were also hybridized with the previously designed *Zoogloea*‐targeting probe, ZOO834 (Figure 1C). Therefore, from the combination of GRA665/GRA686 and ZOO834 probes, we observed most HHAS10‐like cells in dense clusters of *Zoogloea* (Figure 1C,D). Additionally, HHAS10‐like cells were always associated with *Zoogloea* cells, and there were no HHAS10‐like cells associated with other bacteria or free‐living. Some members of Ca. Paceibacteria are known to localize to the cell poles of *Methanospirillum* (Kuroda, Kubota, et al., 2022); however, the HHAS10‐like cells observed in this study did not adhere to specific sites on the *Zoogloea* cells (Figure 1D **)**. *Zoogloea* is known to form dense cell aggregates, such as the formation of activated sludge flocs; when HHAS10‐like cells formed dense clusters, the clusters of zoogloeal cells tended to decrease (Figure 1E–G). Thus, HHAS10 may have a negative effect on *Zoogloea*. Additionally, some of JAEDAM01 were present very close to *Zoogloea* cells that appeared to be undergoing cell division (Figure 1H). Previous studies on Ca. Patescibacteria (He et al., 2015; Kuroda, Yamamoto, et al., 2022; Kuroda, Kubota, et al., 2022) suggest that HHAS10 have symbiotic relationships with other bacteria as hosts. However, further studies are required to clarify HHAS10's effect on the growth condition of *Zoogloea*.

### *The lower FISH signal intensity of* Zoogloea *in the vicinity of without HHAS10*

**FIGURE 2:**
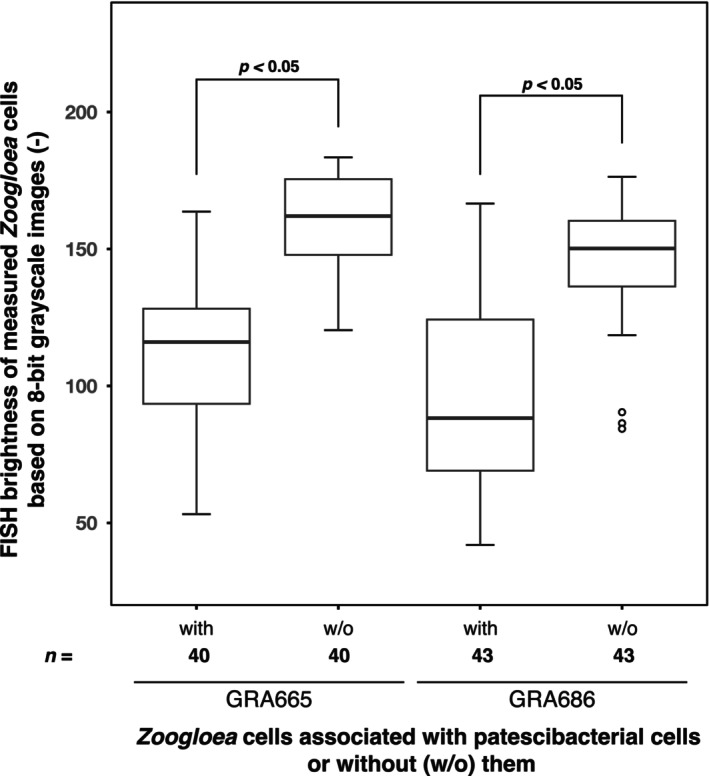
The results of the measuring the brightness values. Boxplots were calculated using ImageJ based on fluorescence in situ hybridization (FISH) signals using GRA665/GRA686 probes targeting JAEDAM01 and ZOO834 probes targeting *Zoogloea*. *n* represents the number of samples. Statistical analysis was performed using Welch's *t*‐test.

In the activated sludge flocs, with and without HHAS10‐like cells, we measured whether the fluorescence signal of *Zoogloea* would be different (Figure 2). When observed with GRA665, the mean fluorescence brightness of cells with HHAS10‐like cells in close association was significantly lower than that of cells without HHAS10 (*p* < 0.05). Approximately the same result was obtained when the GRA686 was used, although there were differences in the mean values of brightness. Ca. Yanofskybacteria, a member of Ca. Patescibacteria is known to physically attach to *Methanothrix*; Ca. Yanofskybacteria attachment decreases the ribosomal activity (based on FISH results) of *Methanothrix*, and their cells often have deformed cell walls, suggesting that the relationship is parasitic (Kuroda et al., 2024; Kuroda, Yamamoto, et al., 2022). These results indicate that HHAS10 have negative effects on the activities of *Zoogloea*, and thus, the parasitic relationship between HHAS10 and *Zoogloea* was speculated. It has been reported that the cell walls of hosts in stable symbiotic relationships with *Patescibacteria* become thicker (Hendrickson et al., 2022). Moreover, in wastewater treatment systems, EPS produced by microbial metabolism is sensitive to environmental conditions (Sheng et al., 2010). It cannot be excluded that the composition of the EPS produced may be changed by external factors, such as parasitization by other bacteria. It should also be noted that these factors may modify the accessibility of the probe to the target.

### *Global distribution of the relationship between JAEDAM01 and* Zoogloea

To confirm the global distribution of the JAEDAM01‐*Zoogloea* associations in the wastewater treatment plants, we analysed 16S rRNA gene sequences of 1069 activated sludge samples recently reported by Hu et al. (2024). Two OTUs (Zoo_OTU1 and Zoo_OTU2) closely related to the 16S rRNA gene sequence of *Zoogloea* EMIRGE01 (>97% identity) were found in many of the wastewater treatment plants worldwide (Figure S2 and datasheet 3 in Data S2). Meanwhile, two OTUs (JAEDAM01_OTU1 and JAEDAM01_OTU2) with >97% identity to the 16S rRNA gene sequence of the MAG HHAS10 were present in 7 and 28 samples, respectively. Although the distributed abundances were scarce, these OTUs were detected from activated sludge samples in 12 countries, and the detected geographical regions were completely disparate (Table S3 and datasheet 3 in Data S2). To further investigate the global distribution of JAEDAM01 microbes, we selected 51 OTUs that perfectly matched the GRA686 probe (datasheet 2 in Data S2). A total of 51 JAEDAM01 OTUs were present in 525 wastewater treatment samples, distributed over 29 countries with 6 regions (Figure S2, datasheets 1 and 2 in Data S2). Global distributions of the relationship between JAEDAM01 and *Zoogloea* were also predicted through Spearman's rank correlation coefficients (Table S4). The results indicate that representative OTUs of *Zoogloea* and JAEDAM01 have significant and weak correlations (*p* < 0.05 and *r*s >0.06), suggesting that JAEDAM01‐*Zoogloea* associations are potentially present in the global wastewater treatment plants. To correctly predict the interactions between JAEDAM01 and *Zoogloea*, further investigations such as a collection of long‐term and time‐series microbiome data along with monitoring of environmental factors may be required (Wang et al., 2021, 2023).

### *Genomic traits and predicted relationship between HHAS10 and* Zoogloea

*Zoogloea* is known to be involved in floc formation in the activated sludge by secretion of extracellular polymeric substance (EPS). In the two reconstructed *Zoogloea* MAGs (Figure S3A), we identified homologues of clusters related to EPS biosynthesis and export and floc formation (An et al., 2016; Gao et al., 2018) (Figure S3B). We identified most of the critical genes (shown as pink in Figure S3B) for each function in the *Zoogloea* MAGs and other functional genes for polyhydroxyalkanoate (PHA) accumulation, denitrification, sulfur metabolism and glycogen accumulation, suggesting that *Zoogloea* may be involved in nutrient removal and floc formation within activated sludge (datasheets 4–12 in Data S2).

**FIGURE 3:**
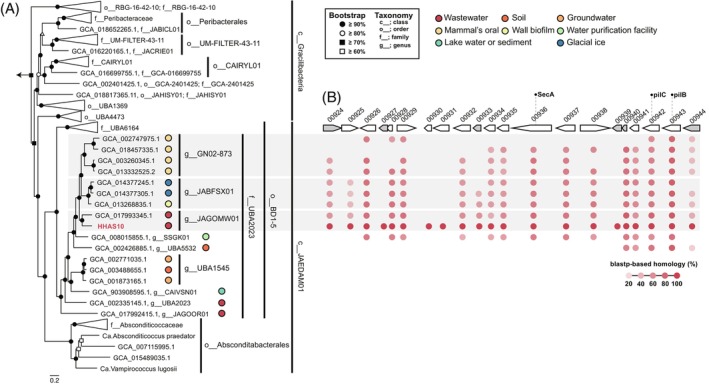
Phylogenetic and cluster genes analysis. (A) Phylogenetic tree of the class Ca. Gracilibacteria and JAEDAM01 based on concatenated phylogenetic marker genes of GTDB‐Tk v2.2.6 (R207). The phylogenetic position of the metagenomic bin is shown in red. (B) Gene clusters containing multiple genes in the metagenome‐assembled genomes (MAGs) in family UBA2023. Red circles indicate a BLASTp‐based homology (threshold ≤1e–10) with HHAS10. No annotated genes or hypothetical proteins are shown in grey.

The reconstructed JAEDAM01 MAG, HHAS10, was classified as c__JAEDAM01, o__BD1‐5 in the GTDB classification (Figure 3A), while c__Gracilibacteria were intermingled inside c__JAEDAM01 on the 16S rRNA gene‐based phylogenetic tree (Figure S4). In the concatenated phylogenetic tree, the closely related species at the genus level were from wastewater; however, those at the family level were not from a specific environment (Figure 3A). Next, we identified genes involved in type IV pili (pilB, ‐C, ‐D and ‐T), which are common in many Ca. Patescibacteria. Genes except for pilD were present in two copies in the genome. Only one copy of pilB and pilC were contiguous in the genome; this trend was the same in closely related species at the family level. The regions before and after those tended to be conserved in closely related species; however, they were completely different from pili function or hypothetical proteins (Figure 3B). Further investigation, such as transcriptomics and proteomics, is necessary to clarify the role of the cluster of its lineage. Type IV pili have recently been shown to be involved in adhesion to host bacteria and host‐specific recognition (Xie et al., 2022) and have been implicated in DNA uptake through conjugation with the competence protein EC (ComEC) gene (Méheust et al., 2019). Because HHAS10 also possesses the *ComEC* gene, it may be involved in these roles (datasheet 13 in Data S2).

Ca. Gracilibacteria has been reported to encode UGA as a glycine instead of the stop codon (Hanke et al., 2014; Rinke et al., 2013). Examination of the genetic code of all MAGs in the phylogenetic tree showed that the stop codon UGA of MAGs belonging to c__JAEDAM01, including HHAS10, was reassigned as a glycine codon. However, the MAGs belonging to c__Gracilibacteria had the UGA as a stop codon as usual (datasheet 14 in Data S2). Another unique feature of HHAS10 is that it possesses pyruvate kinase in its glycolysis pathway. No membrane transporters, such as for phosphoenolpyruvate (PEP), were observed. Thus, whether the HHAS10 incorporates PEP from outside the cell or internally synthesizes it remains to be clarified. As a further feature, HHAS10 possessed four copies of the peptidase of the M23 family. It is known to be a bacteriolytic enzyme, which affects the cell wall and inhibits the growth of sensitive bacteria (Baba & Schneewind, 1996). It is unclear whether *Zoogloea* is sensitive to this peptidase; however, *Zoogloea* might be affected based on the FISH observations and the predominant decreasing trend in fluorescence brightness values.

DNA, polysaccharides and other macromolecules are found in the EPS fraction of activated sludge (Frølund et al., 1996). Because HHAS10 also possesses PHA depolymerase; they could form a symbiotic relationship with *Zoogloea*. Because *Zoogloea* may also be involved in PHA accumulation, they can create an environment favourable for the survival of HHAS10 (datasheet 13 in Data S2).

These findings strongly suggest a novel episymbiotic lifestyle of Ca. Patescibacteria on the *Zoogloea*‐formed flocs and this episymbiotic relationship must be investigated in more detail.

## AUTHOR CONTRIBUTIONS

**Naoki Fujii:** Data curation (equal); investigation (lead); writing – original draft (lead). **Kyohei Kuroda:** Data curation (equal); writing – original draft (equal). **Takashi Narihiro:** Data curation (equal); writing – original draft (equal). **Yoshiteru Aoi:** Conceptualization (equal); writing – original draft (equal). **Noriatsu Ozaki:** Conceptualization (equal). **Akiyoshi Ohashi:** Conceptualization (equal). **Tomonori Kindaichi:** Conceptualization (equal); data curation (equal); funding acquisition (lead); supervision (lead); writing – original draft (equal).

## CONFLICT OF INTEREST STATEMENT

The authors declare no competing interests.

## Supporting information


**Data S1.** Supporting information.

**Data S2.** Supporting information.

## Data Availability

The sequence data of the partial 16S rRNA gene sequence were deposited in the GenBank/EMBL/DDBJ databases under the accession number DRA013509. Metagenomic sequence data were deposited in the DDBJ database under the DDBJ/EMBL/GenBank accession number DRA013531.
