# Supplementary material for: Unique episymbiotic relationship between Candidatus Patescibacteria and Zoogloea in activated sludge flocs at a municipal wastewater treatment plant
Source: Environ Microbiol Rep. 2024 Sep 12;16(5):e70007. doi: 10.1111/1758-2229.70007 (PMC11393006; doi:10.1111/1758-2229.70007)
Supplement: Supplementary file 1 — Data S1. Supporting information. [file EMI4-16-e70007-s001.docx]

**Supplementary Information**

**Supplementary Figures**

**
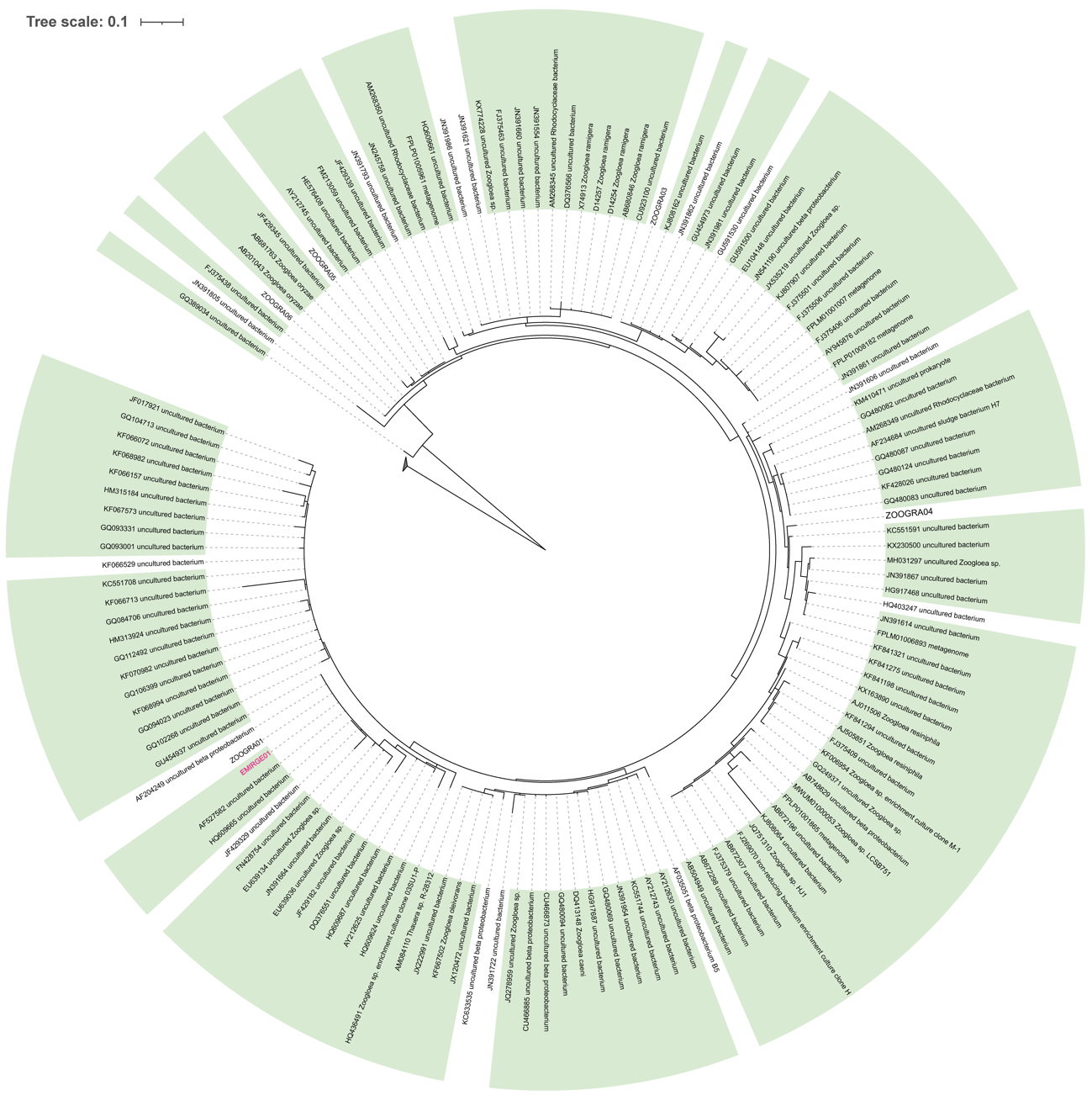
**

**Figure S1.** Maximum likelihood phylogenetic tree of *Zoogloea*-related 16S rRNA genes. Sequences named EMIRGE01 (in pink) indicate 16S rRNA gene reconstructed with EMIRGE software. Sequences named ZOOGRA* were ASVs obtained from 16S rRNA amplicon sequence analysis. The sequences highlighted in green indicate that they may have hybridized with the probe ZOO834 with zero mismatch. 1000-replicate bootstraps were used. Thermotoga sequences were used as outgroup.


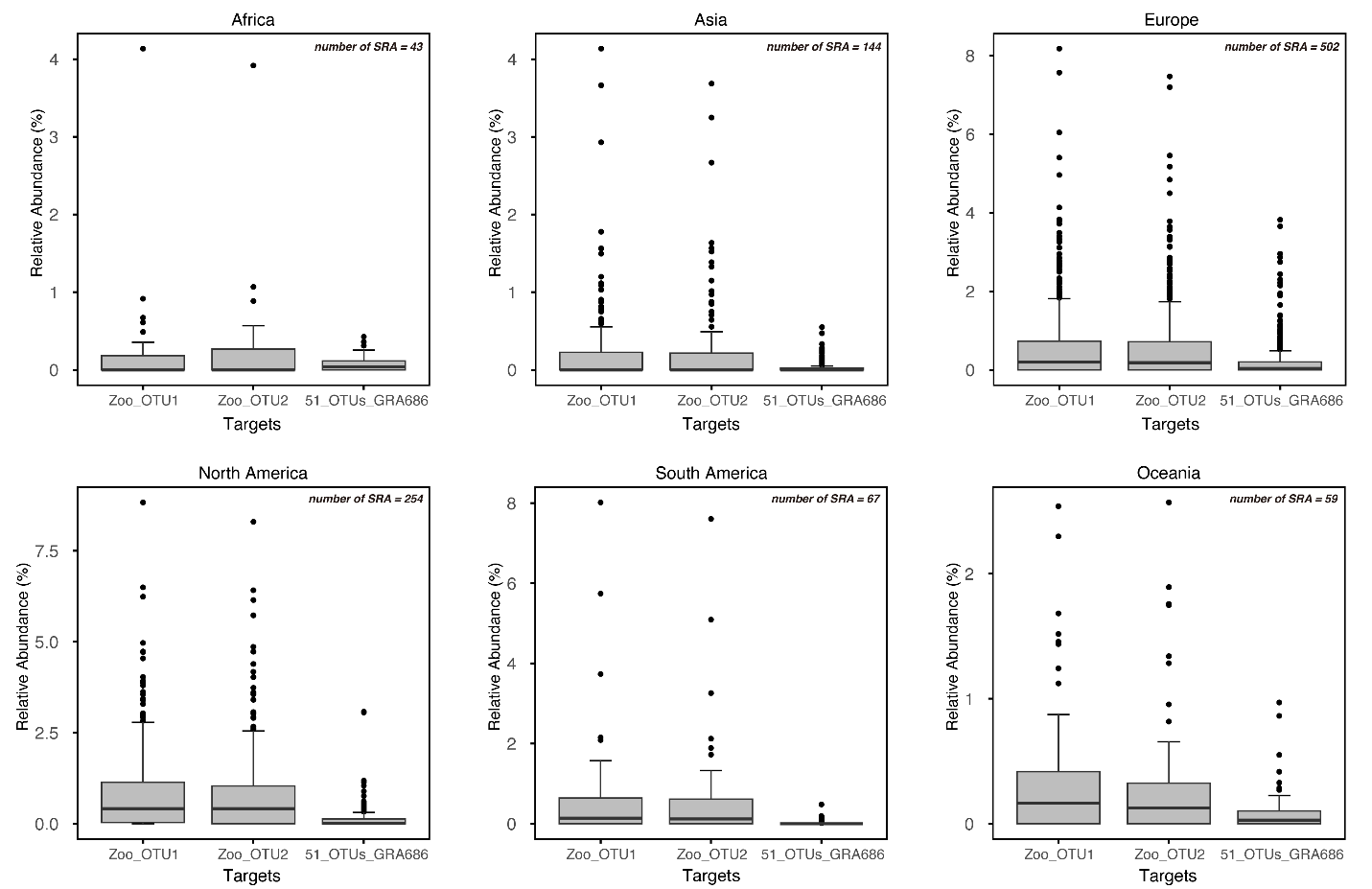


**Figure S2.** The distribution of the relative abundance of Zoo_OTU1, Zoo_OTU2, and 51 OTUs of JAEDAM01 in 1083 samples, for the six regions. The relative abundance of JAEDAM01 in each region is the sum of the 51 OTUs.

**Figure S3.** (A) Phylogenetic tree of the family *Rhodocyclaceae* based on concatenated phylogenetic marker genes of GTDB-Tk v2.2.6 (R207). The phylogenetic position of the metagenomic bins (green). (B) The presence of the extracellular polymeric substance (EPS) biosynthesis cluster genes (16) and phosphoenolpyruvate (PEP)-CTERM proteins (17) in the metagenomic bins. Proteins (in pink) indicate those that are critical in each cluster. Green circles indicate identified genes, and white circles indicate genes could not be identified in this study.


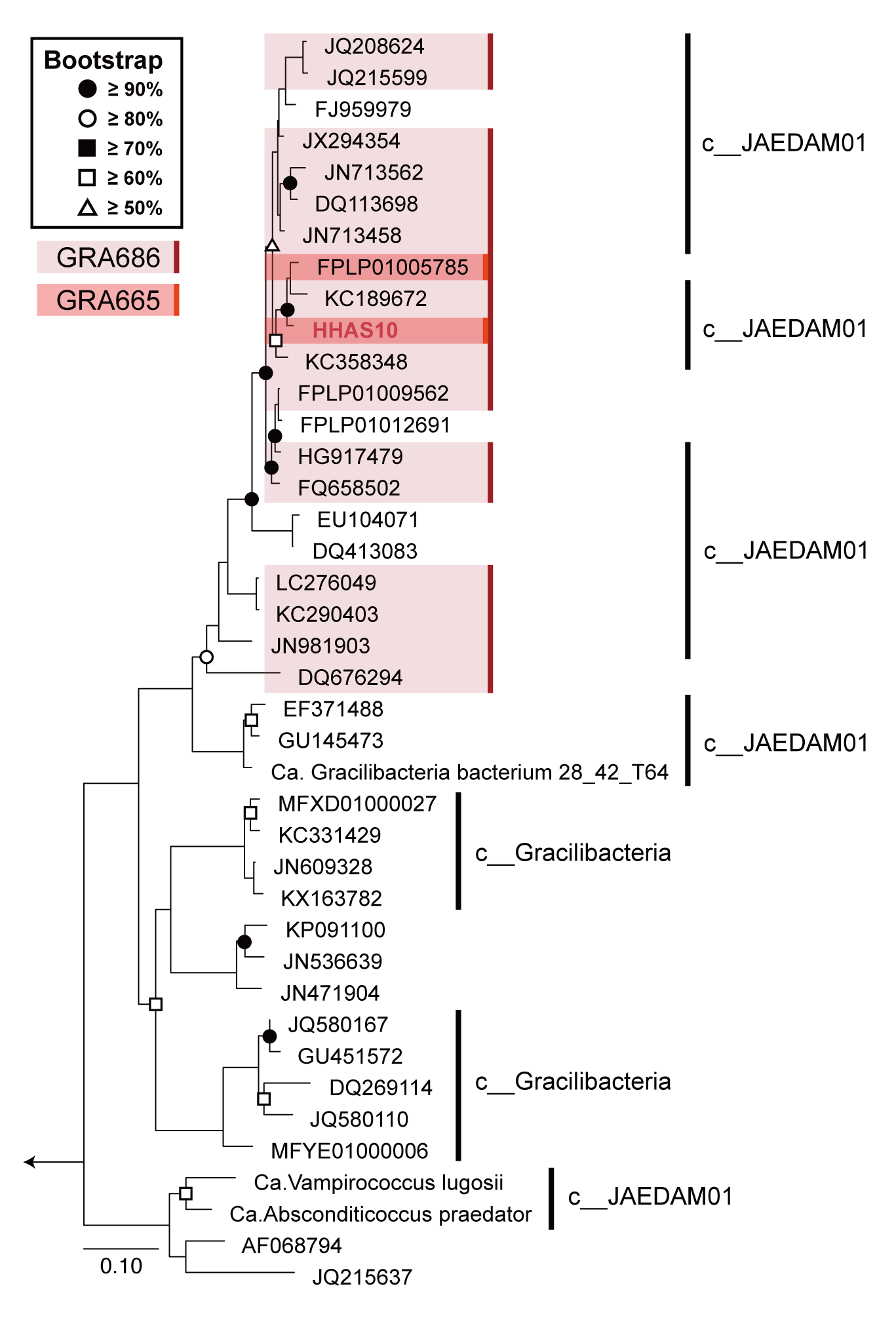


**Figure S4.** Maximum likelihood phylogenetic tree of JAEDAM01- and *Ca.* Gracilibacteria-related 16S rRNA genes. Sequences named HHAS10 (in pink) indicate 16S rRNA genes obtained from the previous metagenomic analysis (1). The vertical, colored lines for the probes GRA665 and GRA686 indicate the sequences possibly hybridized with the probe with zero mismatches. The sequences could not be assigned to a phylogeny are unclassified *Ca.* Patescibacteria.1000-replicate bootstraps were used. Thermotoga sequences were used as outgroup.


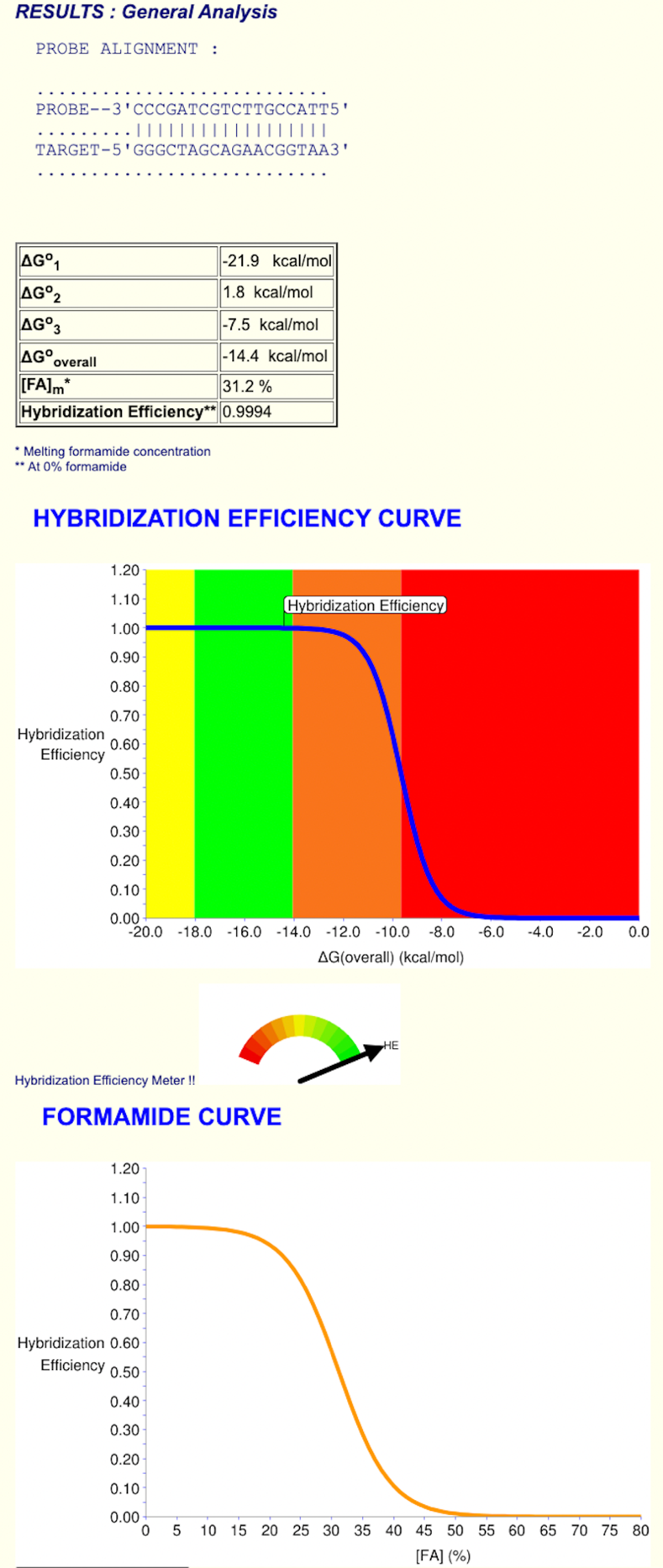


**Figure S5.** mathFISH curve for the GRA665 probe. The target organism is HHAS10.

**
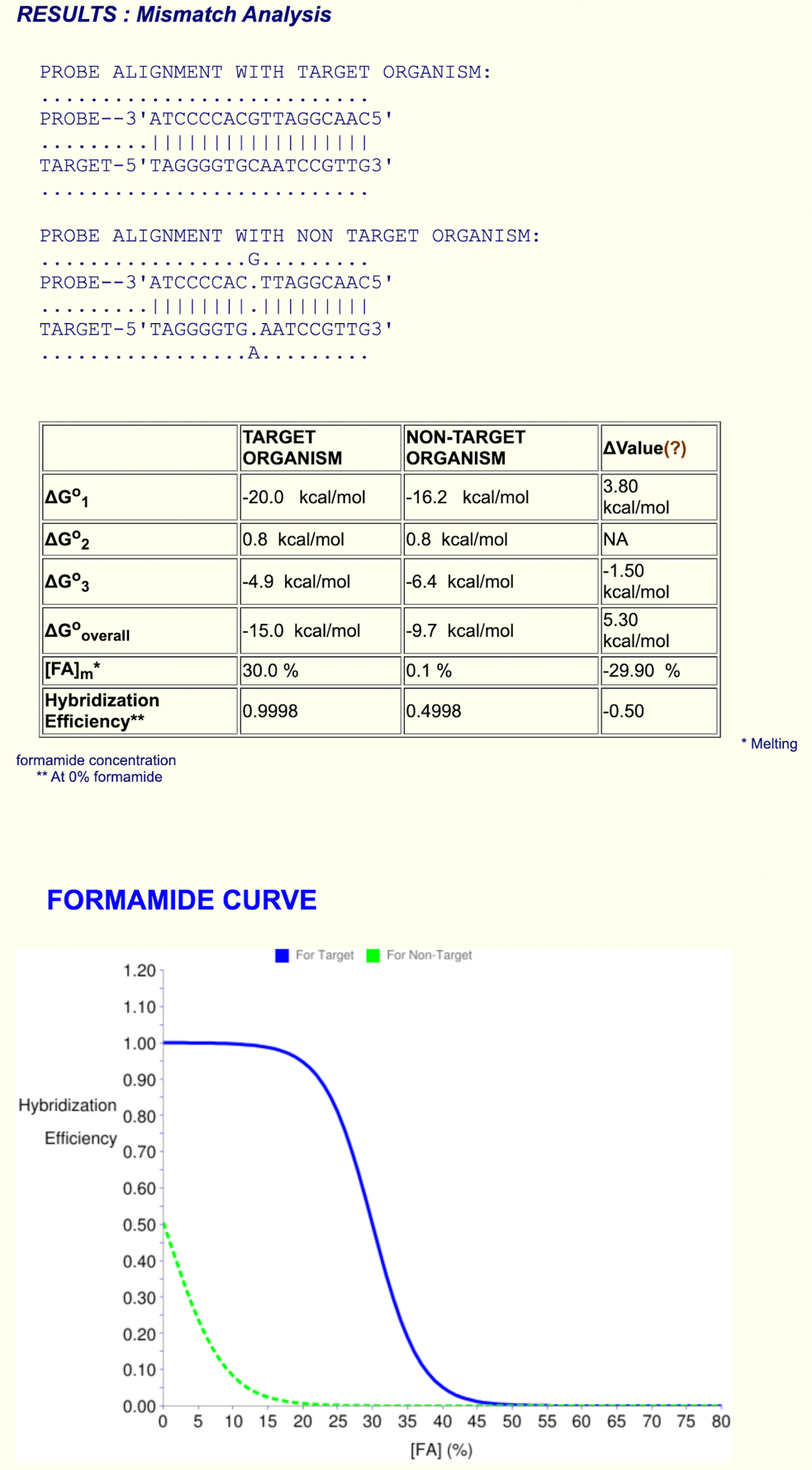
**

**Figure S6**. mathFISH curve for the GRA686 probe. The target organism is HHAS10, and the non-target organism is AY168743.


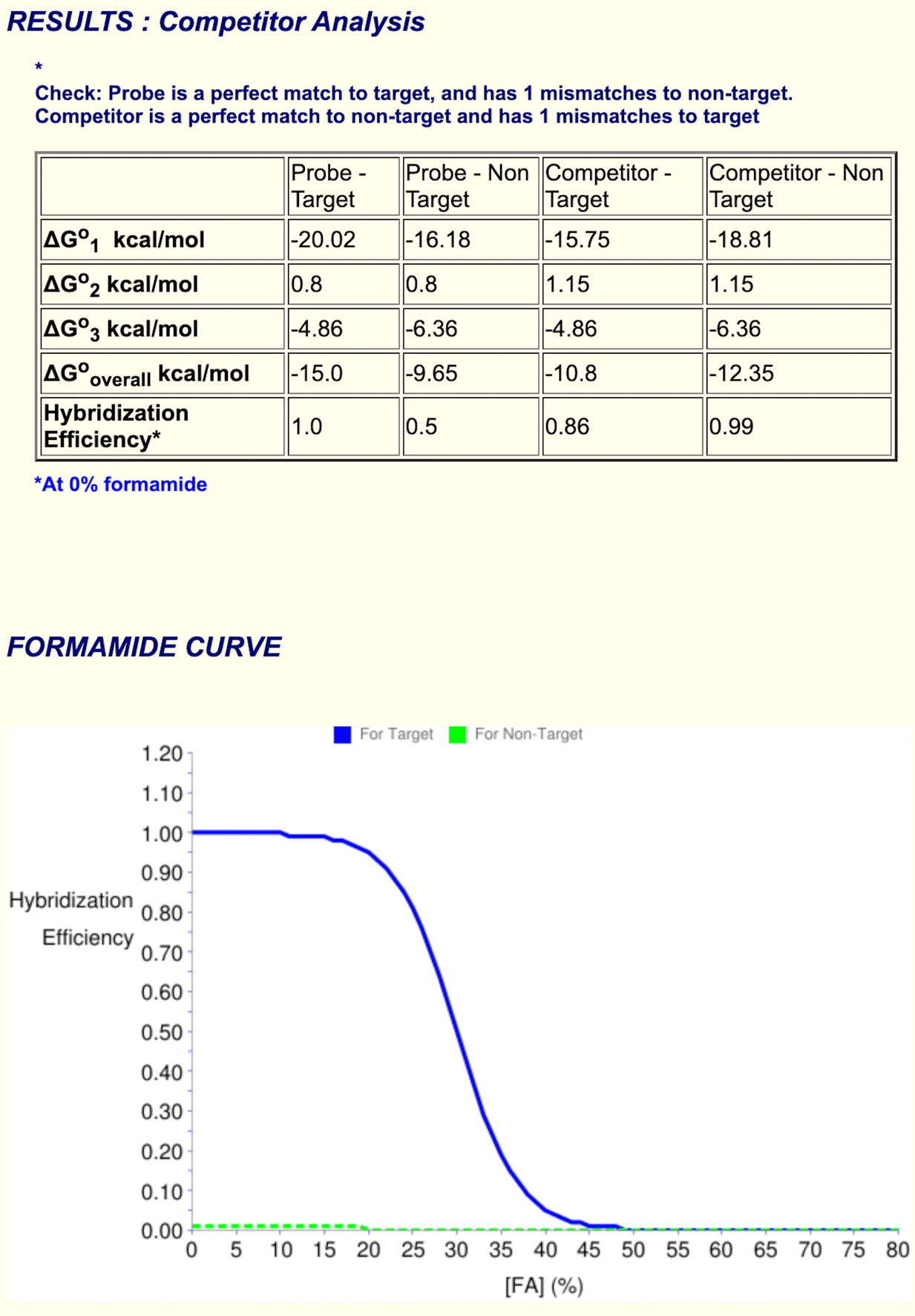


**Figure S7**. mathFISH curve for the GRA686 probe with a competitor probe. The target organism is HHAS10, and the non-target organism is AY168743.


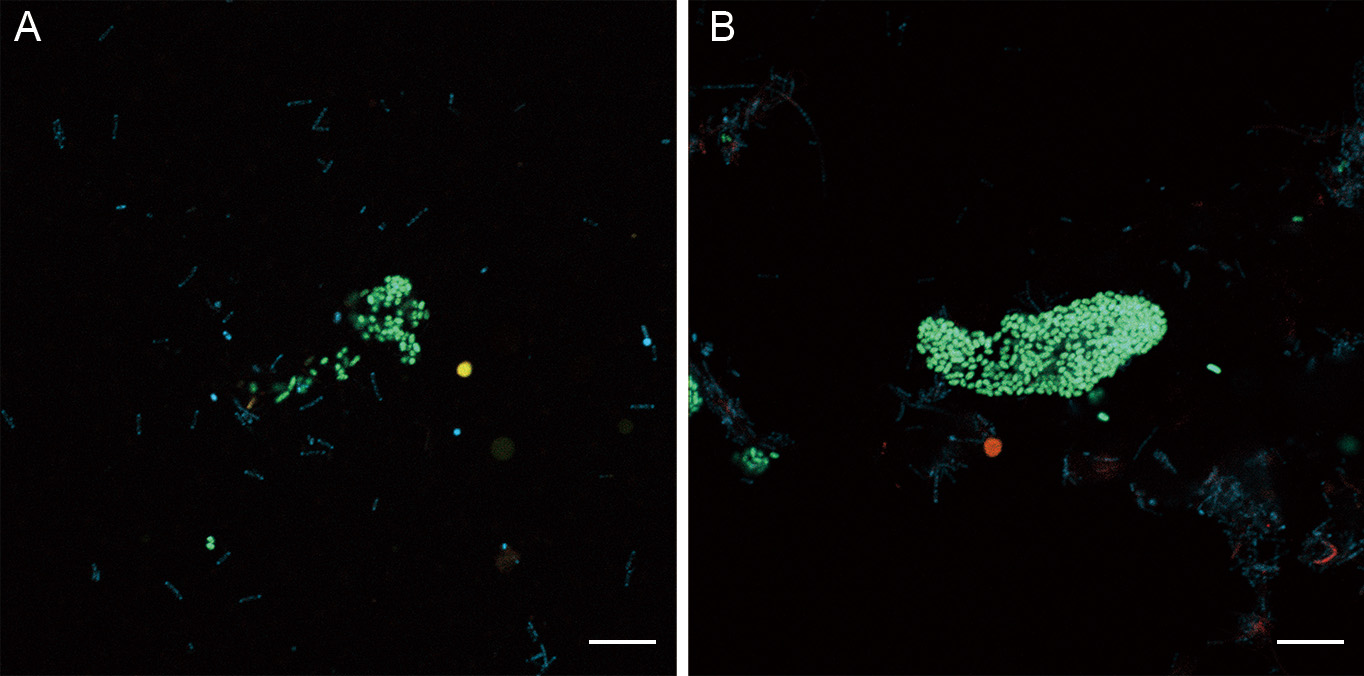


**Figure S8**. Fluorescence *in situ* hybridization (FISH) micrographs of *Zoogloea* with nonsense probes. FISH was performed with three different fluorophores, the Alexa 488-labeled ZOO834 probe (green), Alexa 555-labeled NON-GRA655 (A) or NON-GRA686 probe (B) (red), and Alexa 647-labeled EUBmix probes (blue).


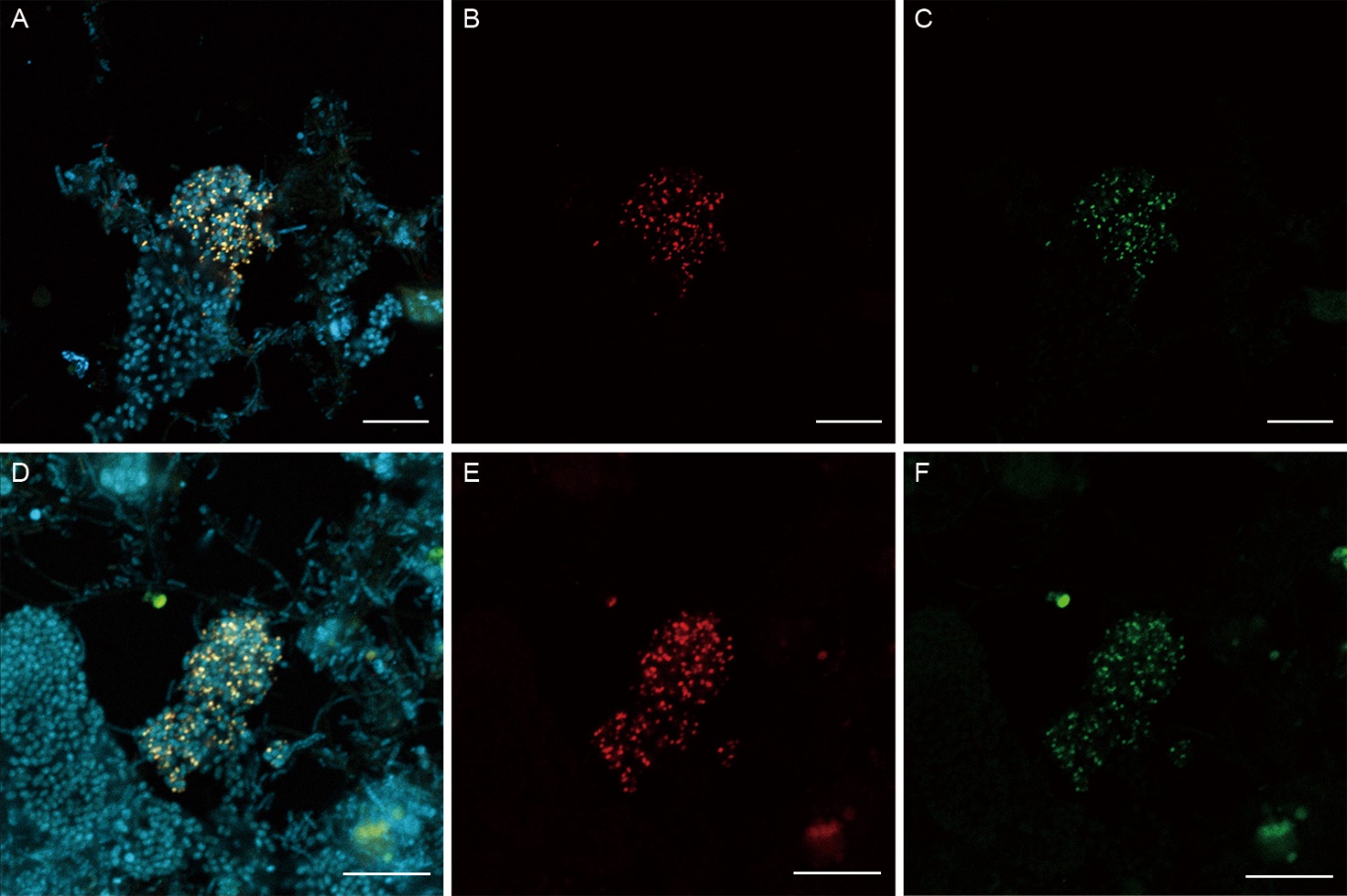


**Figure S9**. FISH micrographs of JAEDAM01. FISH was performed with three different fluorophores, the Alexa 488-labeled GRA686 probe (green), Alexa 555-labeled GRA655 (red), and Alexa 647-labeled EUBmix probes (blue). (A-C) and (D-F) are FISH images of the same field of view, respectively.

**Supplementary datasheet**

Datasheet summarizing the results of the amplicon sequence analysis and the metagenome-assembled genomes of *Zoogloea* and JAEDAM01 annotations.

**Supplementary datasheet 1.** Sample information obtained from the NCBI repository under BioProject accession number PRJNA1013122 (Hu et al., 2024).

**Supplementary datasheet 2.** Information of OTUs belonging to class Gracilibacteria and class JAEDAM01 in global wastewater treatment samples obtained from Hu et al. (2024).

**Supplementary datasheet 3.** Relative abundance of Zoo_OTU1, Zoo_OTU2, JAEDAM01_OTU1, JAEDAM01_OTU2, and sum of 51 OTUs that perfectly matched the newly designed GRA686 probe in each sample.

**Supplementary datasheet 4.** Genes associated with EPS biosynthesis found in MAGs in *Zoogloea* in this study.

**Supplementary datasheet 5.** Results of blastp of genes associated with EPS biosynthesis found in MAGs in *Zoogloea* in this study.

**Supplementary datasheet 6.** Genes associated with PEP-CTERM proteins found in MAGs in *Zoogloea* in this study.

**Supplementary datasheet 7.** Results of blastp of genes associated with PEP-CTERM proteins found in MAGs in *Zoogloea* in this study.

**Supplementary datasheet 8.** Genes associated with Polyphosphate metabolism found in MAGs in *Zoogloea* in this study.

**Supplementary datasheet 9.** Genes associated with glycogen metabolism found in MAGs in *Zoogloea* in this study.

**Supplementary datasheet 10.** Genes associated with PHA metabolism found in MAGs in *Zoogloea* in this study.

**Supplementary datasheet 11.** Genes associated with Sulfur metabolism found in MAGs in *Zoogloea* in this study.

**Supplementary datasheet 12.** Genes associated with Nitrogen metabolism found in MAGs in *Zoogloea* in this study.

**Supplementary datasheet 13.** Genes associated with Polyphosphate utilization, PHA degradation, Pyruvate metabolism, and DNA uptake found in MAG HHAS10 in JAEDAM01 in this study.

**Supplementary datasheet 14.** Results of genetic code estimation for class Gracilibacteria and class JAEDAM01 MAGs on the phylogenetic tree in Fig. 3A.

**Supplementary Tables**

**Table S1.** ASVs of *Zoogloea* obtained using QIIME2. The relative abundance of each sample is shown.

|  | AS201902 (%) | AS202004 (%) | AA202004 (%) |
| --- | --- | --- | --- |
| ZOOGRA01 | 0.06 | 3.82 | 1.58 |
| ZOOGRA03 | 0 | 0.45 | 0.55 |
| ZOOGRA04 | 0 | 0.30 | 0.18 |
| ZOOGRA05 | 0 | 0.05 | 0.19 |
| ZOOGRA06 | 0.02 | 0.13 | 0 |

**Table S2.** Basic information on the reconstructed JAEDAM01 and *Zoogloea* genomes.

|  | **Taxonomy** | **Abundance** | | | **Quality Estimation** | | | **Bin Size** |
| --- | --- | --- | --- | --- | --- | --- | --- | --- |
|  |  | **AS201902** | **AS202004** | **AA202004** | **Completeness** | **Contamination** | **Strain Heterogeneity** | **(Mbp)** |
| HHAS10 | JAEDAM01 | 0.02 | 0.22 | 0.19 | 97.67* | 0* | 0* | 1.3 |
| metagenome1 | *Zoogloea* | 0.39 | 1.94 | 0.64 | 92.48 | 5.03 | 28.57 | 4.5 |
| metagenome2 | *Zoogloea* | 0.02 | 0.39 | 0.31 | 72.41 | 3.45 | 0 | 3.8 |

^*^Calculated using the CPR marker set.

**Table S3.** Relative abundance of Zoo_OTU1, Zoo_OTU2, JAEDAM01_OTU1, and JAEDAM01_OTU2 in 35 samples of activated sludge. The samples contain >0% relative abundance of any of these OTUs.

| Biosample | Region | Country | Zoo_OTU1 | Zoo_OTU2 | JAEDAM01_OTU1 | JAEDAM01_OTU2 |
| --- | --- | --- | --- | --- | --- | --- |
| SRR26542987 | Asia | Singapore | 0.61% | 0.64% | 0.04% | 0.00% |
| SRR26562031 | Europe | Switzerland | 0.60% | 0.77% | 0.16% | 0.00% |
| SRR26543271 | North America | USA | 0.00% | 0.00% | 0.02% | 0.00% |
| SRR26543231 | Oceania | Australia | 1.44% | 1.28% | 0.14% | 0.00% |
| SRR26543241 | Oceania | Australia | 1.24% | 1.34% | 0.06% | 0.00% |
| SRR26561888 | Oceania | Australia | 2.30% | 1.89% | 0.05% | 0.00% |
| SRR26562115 | Oceania | Australia | 1.68% | 1.75% | 0.08% | 0.00% |
| SRR26561916 | Europe | Belgium | 1.95% | 1.95% | 0.00% | 0.06% |
| SRR26562249 | Africa | South Africa | 4.13% | 3.92% | 0.00% | 0.09% |
| SRR26562125 | Europe | United Kingdom | 0.48% | 0.57% | 0.00% | 0.06% |
| SRR26543030 | South America | Argentina | 0.66% | 0.83% | 0.00% | 0.12% |
| SRR26561970 | South America | Argentina | 1.13% | 1.19% | 0.00% | 0.05% |
| SRR26543243 | Oceania | Australia | 0.00% | 0.00% | 0.00% | 0.97% |
| SRR26562117 | Oceania | Australia | 0.00% | 0.00% | 0.00% | 0.16% |
| SRR26562219 | Europe | Sweden | 1.42% | 1.54% | 0.00% | 0.05% |
| SRR26543224 | Europe | Sweden | 0.65% | 0.51% | 0.00% | 0.10% |
| SRR26562202 | Europe | Sweden | 0.00% | 0.00% | 0.00% | 0.05% |
| SRR26561959 | Europe | Austria | 4.13% | 4.50% | 0.00% | 0.05% |
| SRR26543009 | Europe | Finland | 0.81% | 1.22% | 0.00% | 0.03% |
| SRR26562163 | North America | USA | 0.85% | 0.85% | 0.00% | 0.12% |
| SRR26543308 | North America | USA | 0.61% | 0.60% | 0.00% | 0.14% |
| SRR26561781 | North America | USA | 6.50% | 5.73% | 0.00% | 0.04% |
| SRR26561767 | North America | USA | 1.96% | 1.74% | 0.00% | 0.15% |
| SRR26543106 | North America | USA | 1.41% | 1.31% | 0.00% | 0.24% |
| SRR26543104 | North America | USA | 3.80% | 3.61% | 0.00% | 0.06% |
| SRR26561765 | North America | USA | 3.87% | 3.41% | 0.00% | 0.05% |
| SRR26561760 | North America | USA | 3.29% | 3.07% | 0.00% | 0.01% |
| SRR26543405 | North America | USA | 2.68% | 2.91% | 0.00% | 0.12% |
| SRR26561740 | North America | USA | 2.32% | 2.27% | 0.00% | 0.04% |
| SRR26561738 | North America | USA | 1.66% | 1.43% | 0.00% | 0.11% |
| SRR26543399 | North America | USA | 4.72% | 6.42% | 0.00% | 0.09% |
| SRR26561734 | North America | USA | 3.92% | 4.86% | 0.00% | 0.12% |
| SRR26543297 | Europe | Spain | 1.11% | 1.08% | 0.00% | 0.04% |
| SRR26543294 | Europe | Spain | 0.89% | 0.91% | 0.00% | 0.10% |
| SRR26561720 | Europe | Spain | 0.86% | 1.02% | 0.00% | 0.06% |

**Table S4.** Spearman’s rank correlation between JAEDAM01 and *Zoogloea*.

| ASV of Zoogloea | ASV of JAEDAM01 | correlation-value *rs* | p-value |
| --- | --- | --- | --- |
| Zoo_OTU1 | JAEDAM01_OTU1 | 0.069 | 0.025 |
| Zoo_OTU2 | JAEDAM01_OTU1 | 0.072 | 0.019 |
| Zoo_OTU1 | JAEDAM01_OTU2 | 0.174 | 9.8E-09 |
| Zoo_OTU2 | JAEDAM01_OTU2 | 0.19 | 4.3E-09 |
| Zoo_OTU1 | 51_OTUs_GRA686^*^ | 0.15 | 1.6E-06 |
| Zoo_OTU2 | 51_OTUs_GRA686 | 0.15 | 1.2E-06 |

^*^Sum of the relative abundance of 51 OTUs that perfectly matched the newly designed GRA686 probe.
